# Folk knowledge of wild food plants among the tribal communities of Thakht-e-Sulaiman Hills, North-West Pakistan

**DOI:** 10.1186/s13002-016-0090-2

**Published:** 2016-04-08

**Authors:** Khalid Ahmad, Andrea Pieroni

**Affiliations:** Department of Environmental Sciences, COMSATS Institute of Information Technology, Abbottabad, Pakistan; University of Gastronomic Sciences, Piazza Vittorio Emanuele 9, I-12060 Pollenzo, Italy

**Keywords:** Wild Food Plants, Ethnobotany, Thakht-e-Sulaiman Hills, Pakistan

## Abstract

**Background:**

Indigenous communities of the Thakht-e-Sulamian hills reside in the North-West tribal belt of Pakistan, where disadvantaged socio-economic frames, lack of agricultural land and food insecurity represent crucial problems to their survival. Several studies in diverse areas worldwide have pointed out the importance of wild food plants (WFPs) for assuring food sovereignty and food security, and therefore the current study was aimed at documenting traditional knowledge of WFPs and analyzing how this varies among generations.

**Methods:**

Ethnobotanical data were collected during 2010–2012. In total of seventy-two informants were interviewed in ten villages via in-depth interviews and group discussions with key informants followed by freelisting. Data were analyzed through descriptive and inferential statistics and novelty was checked by comparing the gathered data with the published literature.

**Results:**

A total of fifty-one WFP species belonging to twenty-eight families were documented. Rosaceae was the dominant family with the largest number of species and highest frequency of citation (FC). July was the peak month for availability of WFPs, and fruit was the most commonly consumed part. Among the most cited species, *Olea ferrugenia* was ranked first with a FC = 1, followed by *Amaranthus spinosus* (FC = 0.93). Of the documented species about 14 % (7) were marketable and 27 % (14) were reported for the first time to be used as WFP species in Pakistan.

**Conclusion:**

WFPs still play an important role in the food and culture of the study area and the folk knowledge attached to them is remarkable in the region, although declining among the younger generations. The recorded species needs to be re-evaluated in local projects aimed at fostering endogenous strategies of food security, as well as re-evaluating cultural heritage and sustaining small-scale food market circuits.

## Background

A large variety of wild plants are used as food in diverse communities around the globe. These WFPs vary with the surrounding biodiversity and consequently influence food habits [1]. Wild food flora is a vital element of the diet of rural populations. It suits the community of that particular belief and culture as a food because of its traditionally acquired knowledge-based principles, feelings, and manners. Wild collected food plants have been part of the human diet since time immemorial and it has been argued that past societies made more use of wild flora than is done nowadays [2, 3]. Due to their remarkable nutrient values as well as being an excellent source of minerals, fiber, vitamins, and fatty acids that add flavor and color to the diet, WFPs play a key role in complementing staple foods [4–7].

Despite the marked increase in food production, 33 % of mountain people in developing countries are facing hunger, malnutrition and starvation [8]. By the year 2050 the world’s population is expected to reach 9 billion, requiring 70–100 % more food than today. This issue demands substantial attention in order to explore new food assets. The genetic manipulation of crops is considered a potential way to enhance quantity and protect them from diseases and various stresses [9]. WFPs have considerable potential for the development of new crops through domestication and provide a genetic reserve for hybridization and selection [10, 11]. Furthermore, previous ethnobotanical surveys indicate that more than 7000 species are being used by humans as food and for livelihood in poor communities [12, 13].

At present, human populations rely on a small number of cultivated species, domesticated during the past 13,000 years that partly reduced both the beneficial and toxic effects of secondary plant metabolites [14, 15]. In contrast, the human genome cannot fully adjust to the intense changes in diet and lifestyle, which is still adapted to the pre-agriculturalist diet pattern. Modern lifestyle diseases are due to alterations in major functional dietary components which were endowed with prophylactic effects in wild gathered food [3, 15–19]. Some important WFP knowledge is restricted to particular communities but such specific knowledge decreases quite rapidly due to its fragile nature. Therefore, ethnobotanical field research for documentation and evaluation of this traditional knowledge is of great importance [20] in providing insight into food diversification which must be identified.

Pakistan is a developing country with an area of about 10 million hectares (0 – 8611 m altitude), which has different climatic zones–tropical, subtropical and temperate –featuring a unique and vast floral diversity that harbors more than 6000 vascular plant species [21–23]. Despite the various climatic zones and floral diversity, Pakistan is ranked 11^th^in the Food Security Risk Index [24]. An alarming situation is presented by the National Nutrition Survey of Pakistan [25] which stated that almost 58 % households are food insecure. Furthermore, the WHO reported in 2010 that 50 % of child deaths are directly or indirectly due to malnutrition in Pakistan [26]. The availability of WFPs in different seasons becomes more important when cultivated fruits and vegetables are not available.

In spite of their great importance, WFPs are vanishing from traditional diets, which poses serious concerns due to their role and contribution in the cultural history of a region as well as their nutraceutical value [27]. In the developing world these plants are regularly ignored in governmental policies, agricultural research and extension programs. Over the past decade, the majority of tribal communities on the north-western boarder of Pakistan have been affected by the ‘war on terror’, which has destabilized their traditional knowledge systems. The present research area is semi-arid and mountainous with deficient agricultural land. The people live in extreme poverty with widespread food insecurity. They are also not considered in government developmental policies.

The present study focused on these remote indigenous communities of the Thakht-e-Sulaiman hills, NW Pakistan, to document their knowledge and practices concerning WFPs, which have not been previously explored. The specific aims of this study were:

- to document folk knowledge regarding WFPs in the study area;
- to determine how this knowledge is distributed between locations at lower and higher elevations and among generations in the study area;
- to compare the collected data with the overall Pakistani ethnobotanical literature, in order to possibly identify novel wild food plant records.

## Methods

### Study area and ethnic background

**Fig. 1:**
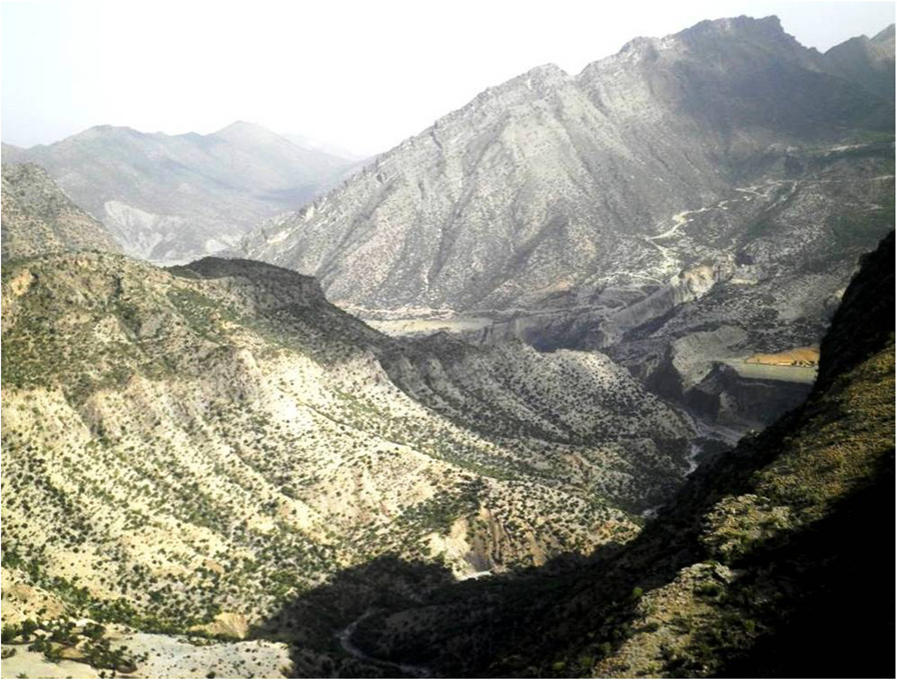
Landscape of the study area

**Fig. 2:**
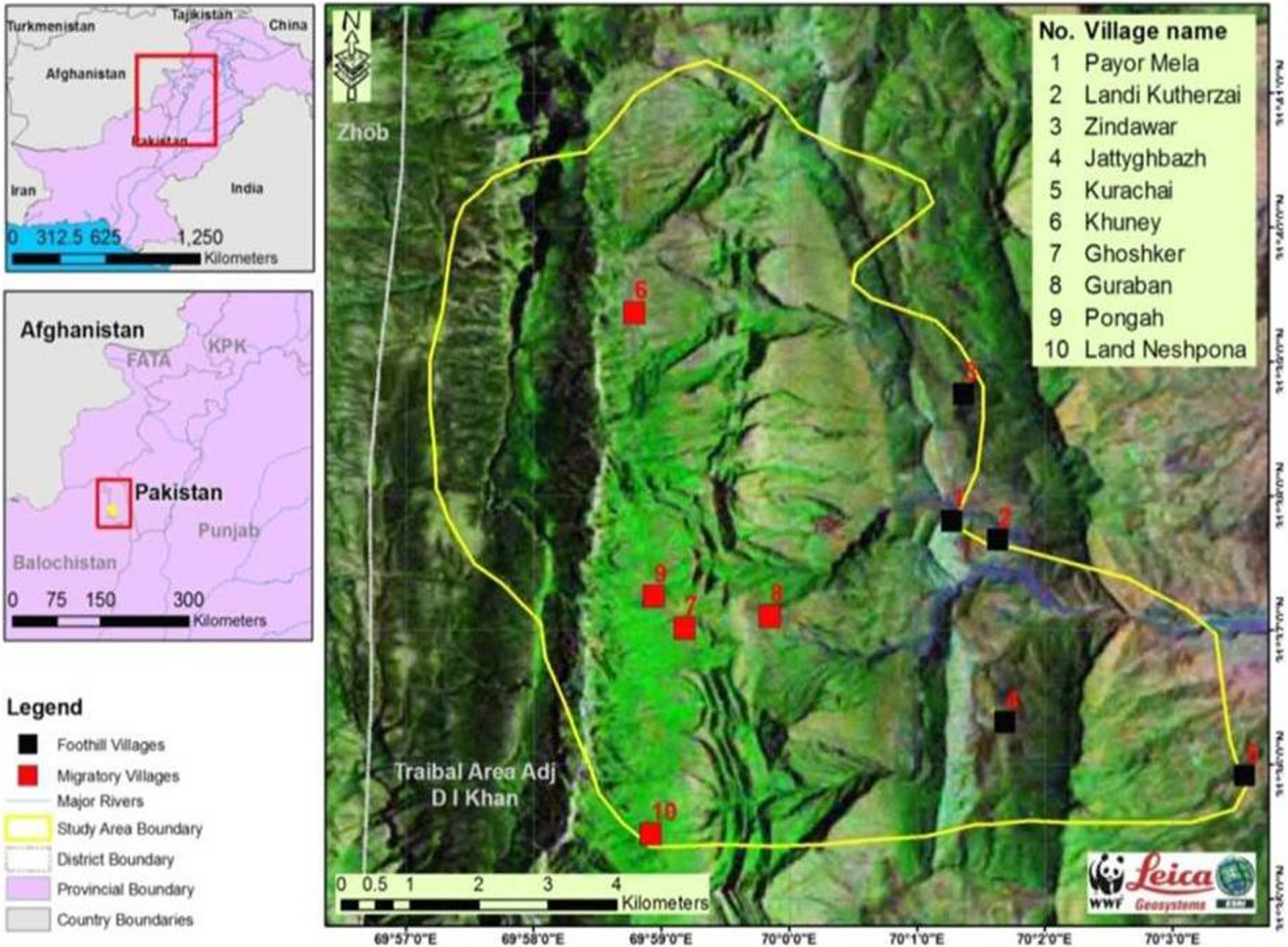
Map of the study area (GPS points were recorded during the fieldwork; WWF Lab, Islamabad, Pakistan)

The Sulaiman mountain range is a geologically important area which forms a border between the Iranian Plateau and the Asian subcontinent. As the highest Plateau in the west and southwest, this range acts as a natural barrier to humid winds coming from the Indian Ocean thus creating arid conditions across southern Afghanistan. Centrally, the Sulaiman range lies a little east of 70° E longitude, and it is geologically composed of great folds of the Cretaceous series. The top of the mountain range is called Thakht-e-Sulaiman–locally known as Kaesagher. The present study was based on the ethnobotanical analysis of WFPs present on the eastern side of the Thakht-e-sulaiman Hills (Fig. 1). This area is situated in the north-west (NW) of Pakistan and serves as a border between the Frontier Region of Dera Ismail Khan (F.R, D.I.Khan) and the Zhobe District of Baluchistan Province. In particular, the study area is encompassed by South Waziristan Agency (north), D.I. Khan (east), Dera Ghazi Khan (south) and Zhob (west). The area is a small administrative unit under Federally Administered Tribal Areas (FATA) of Pakistan (Fig. 2) and two tribes, Sherani and Ustranas, inhabit this frontier region [28]. The Sherani area is totally under the eastern shadow of the highest peak of Thakht-e-Sulaiman. These mountains are covered by coniferous forest and exhibit arid to semi-arid lands that receive 200–500 mm of bimodal precipitation annually [29]. The region experiences hot summers (June, July and August) in which the temperature reaches 40 °C with a monthly rainfall of 21–33 mm and cold winters (December, January and February) in the range of 5.7–7.6 °C with a monthly rainfall of 13 mm [30].

### Field methods

Ethnobotanical field work was conducted for a period of two years during 2010–2012. The study area was divided in two portions based on their altitude: we defined as *foothill villages* those villages located between 750 and 1,800 m.a.s.l., while *mountain/migratory villages* were those villages located between 1,800 and 3,000 m.a.s.l. A total of seventy-two informants (sixty –two men and ten women) from ten different villages were interviewed on the traditional uses of WFPs (including wild fruits, wild vegetables and wild tea species). The ages of the interviewees ranged from 20 to 90 years with an average of 41.8 ± 14.5 years. Detailed unstructured and semi-structured, formal and informal interviews were performed with key informants and group discussions were conducted during the first phase of the study [31]. The first phase was designed to help understand the general perceptions of the locals about natural phenomena [32], to become familiar with ethnographic terms and their emic definitions, and in particular to learn about their interactions with local flora. In the second phase of data collection, successive ‘oral freelisting’ sessions were performed to obtain more salient species and to check disparities in individual and village-wise knowledge. Detailed structured interviews were mostly followed by freelisting. We provided supplementary prompting to enlarge the freelisting [33]. Special care was taken to avoid non-genuine information [34, 35] and responses were cross-checked through informal methods for confirmation. Consent was taken from each informant before every interview and the objectives and procedures of the project were clearly explained. The local language (Pashtu) was used to conduct the interviews with informants, which the first author was able to properly understand. We maintained a continuing relationship with the local communities in order to develop a sense of trust and to be allowed to stay within the foothill and migratory villages and to accompany the locals during their daily activities and to participate in their ceremonies. National and international laws, especially the rules of the Convention on Biological Diversity (CBD) and the ethical guidelines of the International Society of Ethnobiology [36] were strictly followed.

### Sample

An informant from nearly every 3^rd^ house was included in the sample (interview) with minor variations. The population and demographic details of the five foothill villages are shown in Table 1. The locals of two foothill villages (i.e. Payor Mela and Landi Kutherzai) migrate to the high mountains in summer. So, the mountain/migratory villages were based on the residents from these foothill villages while other inhabitants of those villages were from low altitude areas (not included in the study). The names, altitude, and number of houses of each foothill and mountain/migratory village are given in Table 2.

**Table 1:** Social characteristics of the sample and study sites

Village name	PayorMela	LandiKutherzai	Zindawar	JattyGhbaz	Kurachai
Village size (number of families)	36	55	21	42	25
Sample size (number of informants)	18	22	5	18	9
Dependency on livestock as a source of income	75 %	64 %	87 %	55 %	67 %
Informants average age	48.0 ± 18.6	40.9 ± 13.7	36.0 ± 6.5	41.3 ± 12.6	36.7 ± 12.2
Average number of family members	14.9 ± 5.6	12.4 ± 6.6	15.6 ± 6.3	14.7 ± 6.2	10.0 ± 2.4
^a^Migration ratio	50 % (to mountains)	61 % (to mountains)	50 % (within foothills)	38 % (within foothills)	0
Bilingualism	54 %	25 %	1 %	24 %	8 %
Average/month/head expenditure (in PKR)	931 ± 212	1063 ± 370	941 ± 194	1166 ± 537	1420 ± 457

(^a^): in two villages people migrate to the mountains, while in two other villages inhabitants migrate within the foothills area during the summer season; a migration ratio of 50 % indicates that half of the families of a village migrate in summer

**Table 2:** Geographic characteristics of the study sites

Village name	Elevation (m.a.s.l.)	Number of households	Soil, area, population, and vegetation
PayorMela	1116	36	The village has stony soil and thin vegetation within the immediate surroundings, but there is a reserved forest and a very fertile valley in the vicinity; population and area of the village are moderate in size.
LandiKutherzai	1052	55	The village contains fertile loamy soil that can support dense and diverse vegetation; it has a large population and a small area that make the surroundings of the village nearly barren; there are no reserve forests nearby.
Zindawar	1146	21	The village contains various types of soil and a diverse flora. It has a small population and a large area; the vegetation is dense and comparatively undisturbed; it also exhibits floral elements of higher altitudes (mountains).
JattyGhbaz	1155	45	The village contains stony soil; the village population is moderately sized but the area is large; the vegetation is relatively dense.
Kurachai	1184	25	The village has mostly stony soil, which cannot support herbaceous flora; it has a restricted area and a modest population; the vegetation is under anthropogenic pressure.
Khuney	2384	16	It exhibits various types of soils and a diverse flora; the village has a large area and a small population; the density and diversity of the surrounding vegetation is moderate.
Goraban	2456	26	The village contains fertile loamy soil; it has a small area and a modest population; the village is located in a type of valley, which shows diverse vegetations.
Ghoshker	2301	37	The village contains mostly stony soil, which cannot support herbaceous flora. It has a moderate area but a large population; the surrounding vegetation is not dense and is under anthropogenic pressure.
Pongah	2562	40	The village possesses various types of soils which supports a diverse flora; it has a modest area but a large population; the surrounding vegetation is moderately dense and diverse.
Land Nishpona	2584	22	The village contains loamy soil and a diverse and dense flora; it has a small population and a large area; the surrounding vegetation is dense and undisturbed.

(Note: The first five villages are *foothill villages* while the last five villages are *mountain/migratory villages*)

### Collection and identification of plant specimens

Plant specimens were collected in triplicate according to standard botanical and ethnobotanical protocols [37]. The specimens were identified by taxonomists at Quaid-i-Azam University, Islamabad, Pakistan and confirmed by matching their taxonomic characters with the Flora of Pakistan [38, 39]. Family names were assigned according to the Angiosperm Phylogeny Group [40] while species names were assigned in accordance with ‘The Plant List’ [41]. Voucher specimens were numbered and deposited in the Herbarium of Pakistan (ISL) for future reference work.

### Data analysis

Freelisting data were analyzed through descriptive statistics to determine more salient species and informant consensus, using Excel spreadsheets. To check the effect of variables on diversity and variation in folk plant knowledge of individuals and villages, pivot tables were used. T-tests were performed to examine the significance of differences among different categories, while Mann–Whitney-U-Tests for independent samples were performed to establish the distribution of FC values among different groups. FC was determined by counting the number of informants that cited the use of the species divided by the total number of informants that participated in the study. To compare the present data with the published literature from the country, information was gathered from online sources using Google Scholar and Web of Sciences as search engines.

## Results and discussion

### Documentation of wild food plants

Ethnobotanical data of WFPs including vernacular names, parts used, gathering period, gathering areas, collectors and folk food uses are reported in Table 3. A total of fifty-one WFPs from forty-two genera and 28 families were documented. The family Rosaceae was dominant with the largest number of species (7 spp.), followed by Asteraceae (4 spp.), while Apocynaceae, Malvaceae and Rhamnaceae were represented by 3 species each. Based on FC values, the dominant family was Rosaceae followed by Oleaceae, whereas the remaining families with high FC values included Pinaceae, Alliaceae and Moraceae. Most of these taxa are widespread and typically used as wild foods, which have been previously reported in different Eurasian countries [42–46]. However, a rather uncommon food taxon is Apocynaceae– a family known for its toxicity and medicinal potential. *Olea ferrugenia*, *Amaranthus spinosus,* and *Ficus palmata* exhibited the highest FC values (1.0, 0.93 and 0.69, respectively), whereas in other species FC ranged from 0.59 to 0.01. These and related species have been reported by multiple authors from different parts of the country [42, 47–54]. Fruits were the most commonly consumed plant parts (31 spp.) followed by leaves (12 spp.), a finding similar to earlier studies [11, 42]. Regarding growth habit, herbs were dominant (43 %) followed by shrubs, trees and climbers (31, 20 and 6 % respectively). The number of species for each growth habit were significantly different, but the FC values of tree species were the highest followed by shrubs.

**Table 3:** Folk uses of wild food plants recorded in the study area

Botanical taxon, voucher specimen code and botanical family	Folk name	Used part(s)	Gathering period	Gathering area(s)	Collectors	Folk food uses	Citation frequency	Previously reported from Pakistan as WFPs
*Aerva javanica* (Burm.f.) Juss. ex Schult. (127216)	Sparokai	Leaves	April-July	Waste land	Women	Young fresh leaves are boiled in water for ½ hour. The extra water is poured out, and the leaves are moved to another container containing ghee with fried onions & red chilies. Kept on the fire until water is evaporated & ghee appears on top. This is then eaten with bread.	0.16	Yes
Amaranthaceae								
*Allium carolinianum* DC. (127194)	Khokhai	Whole plant	June-September	Ridges	Men	The leaves and bulbs are eaten with bread.	0.37	Yes
Amaryllidaceae								
*Allium* sp. Linn. (127217)	Cook	Whole plant	June-September	Slopes and ridges	Men	The leaves and bulbs are eaten with bread and are also used as spice in curry.	0.50	Yes
Amaryllidaceae								
*Amaranthus spinosus* L. (127219)	Sarmay	Leaves	April-July	Waste land	Women	Young fresh leaves are boiled in water for ½ hour. The extra water is poured out, and then the leaves are moved to another container containing ghee with fried onions & red chilies. Kept on the fire until water is evaporated & ghee appears on top. This is then eaten with bread.	0.93	Yes
Amaranthaceae								
*Berberis calliobotrys* Bien. ex Koehne (127222)	Sarmay	Fruits	October	Pine forests	Men	Directly consumed.	0.11	No
Berberidaceae								
*Caragana ambigua* Stocks (127205)	Zaray	Flowers	June	Valleys	Kids	Directly consumed.	0.02	No
Fabaceae								
*Caralluma tuberculata* N.E.Br. (127133)	Pamanai	Stems	March-April	Moist shady places	Women, men, kids	Extensive salt is rubbed on the cut pieces and left for half an hour. They are then washed with water. Now, they are fried in hot boiling ghee containing fried onions. This is then eaten with bread.	0.22	Yes
Apocynaceae								
*Celtis australis* L. (127176)	Thaghah	Fruits	May	Near human settlements	Men, kids	Directly consumed.	0.05	Yes
Cannabaceae								
*Cicer nuristanicum* Kitam. (127183)	Chenrah	Fruit	July	High mountain valleys	Kids	Directly consumed.	0.06	No
Fabaceae								
*Cirsium arvense* (L.) Scop. (127231)	Da khwarak azghai	Stems	May-August	Waste land	Men, kids	The ectoderm of the semi-mature stem is removed. The remaining white fleshy part is eaten raw.	0.06	No
Asteraceae								
*Cotoneaster microphyllus* Wall. Ex Lindl. (127234)	Manray	Fruit	May	Bushy vegetation	Men, kids	Directly consumed.	0.30	Yes
Rosaceae								
*Cotoneaster minutus* Klotz (127125)	Sharavo	Fruit	September	Shrubby mountain vegetation	Men, kids	Directly consumed.	0.59	Yes
Rosaceae								
*Cotoneaster pruinosus* Klotz (127206)	Pushthawergai	Fruit	August	Beside pedestrian passes	Kids	Directly consumed.	0.22	No
Rosaceae								
*Cynoglossum lanceolatum* Forssk. (127212)	Jezgai	Leaves	April-July	Waste land	Women	Young fresh leaves are boiled in water for ½ hour. The extra water is poured out and then the leaves are moved to another container containing ghee with fried onions & red chilies. Kept on the fire until water is evaporated & ghee appears on top. This is then eaten with bread.	0.01	No
Boraginaceae								
*Debregeasia saeneb* (Forssk.) Hepper & J.R.I.Wood (127143)	Mermandai	Fruit	May	High mountain valleys	Men, kids	Directly consumed.	0.19	Yes
Urticaceae								
*Ficus palmata* Forssk. (127240)	Injar	Leaves, Fruit	April (Leaves), July (Fruit)	Near human settlements	Women, men, kids	The fruits are directly consumed. The young fresh leaves are boiled in water for 2 hours to soften completely. This is then eaten directly or sometimes with bread.	0.69	Yes
Moraceae								
*Grewia tenax* (Forssk.) Fiori (127126)	Pasthawnai	Fruit	August	Hedgerows	Kids	Directly consumed.	0.01	Yes
Malvaceae								
*Grewia villosa* Willd. (127243)	Injarai	Fruit	July	Marshy & bushy vegetation	Kids	Directly consumed.	0.08	Yes
Malvaceae								
*Lactuca dissecta* D.Don (127184)	Paywerka	Leaves	April-August	Hedgerows	Kids	Directly consumed.	0.05	No
Asteraceae								
*Launaea procumbens* (Roxb.) Ramayya & Rajagopal (127180)	Sondrashi	Leaves	April-August	Waste land	Men, Kids	Directly consumed.	0.12	Yes
Asteraceae								
*Limonium cabulicum* (Boiss.) Kuntze (127170)	Botyarai	Leaves	June-August	Rocky barren land	Men	The fresh leaves are boiled in water containing sugar. This is then taken as tea.	0.05	No
Plumbaginaceae								
*Malva sylvestris* L. (127123)	Methrai	Leaves	April-July	Waste land	Women	Young fresh leaves are boiled in water for ½ hour. The extra water is poured out, and then the leaves are moved to another container containing ghee with fried onions & red chilies. Kept on the fire until water is evaporated & ghee appears on top. This is then eaten with bread.	0.08	Yes
Malvaceae								
*Notholirion thomsonianum* (Royle) Stapf (127166)	Shyajey	Leaves	April-May	Sandy fertile places	Women, men	Young fresh leaves are boiled in water for ½ hour. The extra water is poured out, and then the leaves are moved to another container containing ghee with fried onions & red chilies. Kept on the fire until water is evaporated & ghee appears on top. This is then eaten with bread.	0.31	No
Liliaceae								
*Olea ferruginea* Wall. ex Aitch. (127151)	Shwawan	Fruit, Leaves	September	Forests	Women, men, kids	The fruits are directly consumed while the fresh leaves are used as tea.	1.0	Yes
Oleaceae								
*Oxalis corniculata* L. (127198)	Therwashka	Leaves	May	Waste land	Kids	Directly consumed.	0.06	Yes
Oxalidaceae								
*Periploca aphylla* Decne. (127156)	Barrarr	Fruit and young stems	May	Waste land	Kids	The fresh green fruits and young stems are chewed and the sweet tasting sap is swallowed.	0.06	Yes
Apocynaceae								
*Periploca hydaspidis* Falc. (127128)	Khwaza waley	Fruit	May	Barren land	Kids	Directly consumed.	0.01	Yes
Apocynaceae								
*Physalis divaricata* D. Don (127181)	Band malkhovj	Fruit	August	Waste land	Kids	Directly consumed.	0.01	No
Solanaceae								
*Pinus gerardiana* Wall. ex D.Don (127203)	Zanghozai	Fruit	October	Pine forests	Men, kids	Directly consumed.	0.59	Yes
Pinaceae								
*Pinus wallichiana* A.B.Jacks. (127213)	Nashtar	Fruit	October	Pine forests	Men, kids	Directly consumed.	0.29	Yes
Pinaceae								
*Pistacia chinensis* Bunge (127191)	Shrawan	Fruit	October	Forests	Women, men, kids	Directly consumed.	0.30	Yes
Anacardiaceae								
*Pistacia khinjuk* Stocks (127259)	Saho shrawan	Fruit	October	Forests	Men, kids	Directly consumed.	0.01	Yes
Anacardiaceae								
*Portulaca quadrifida* L. (127257)	Pakharai	Aerial parts	May-July	Waste land	Women	Young fresh leaves including young shoots are boiled in water for ½ hour. The extra water is poured out, and then the leaves are moved to another container containing ghee with fried onions & red chilies. Kept on the fire until water is evaporated & ghee appears on top. This is then eaten with bread.	0.04	Yes
Portulacaceae								
*Punica granatum* L. (127208)	Nargos	Fruit	September	Mountains & home gardens	Women, men, kids	Directly consumed.	0.55	Yes
Lythraceae								
*Pyracantha* M. Roam (127127)	Khra sharavo	Fruit	September	Mountain forests	Men, kids	Directly consumed.	0.01	Yes
Rosaceae								
*Ribes alpestre* Wall. ex Decne. (127193)	Sheen korai	Fruit	July	Bushy vegetation	Kids	Directly consumed.	0.01	Yes
Grossulariaceae								
*Rosa moschata* Herrm. (127134)	Khorach	Fruit	August	High mountain valleys	Men, kids	Directly consumed.	0.33	Yes
Rosaceae								
*Rubus ulmifolius* Schott (127197)	Gharangavo	Fruit	August	Fertile mountain valleys	Men, kids	Directly consumed.	0.26	Yes
Rosaceae								
*Salvia moorcroftiana* Wall. ex Benth. (127131)	Dersai	Stem	May-June	Waste land	Women, men, kids	The external green part of the semi-matured stem is removed and the remaining white juicy part is eaten raw.	0.47	No
Lamiaceae								
*Sideroxylon mascatense* (A.DC.) T.D.Penn. (127185)	Guargur	Fruit	June	Low land forests	Women, men, kids	Directly consumed.	0.55	Yes
Sapotaceae								
*Solanum americanum* Mill. (127158)	Malkhovj	Fruit	June	Waste land	Kids	Directly consumed.	0.09	Yes
Solanaceae								
*Spiraea canescens* D.Don (127265)	Sra wany	Fruit, stem, bark	May	Sloppy &shrubby vegetation	Men, kids	The fruits are directly consumed. The green stem cortex is boiled in water with sugar which is then taken as tea	0.12	Yes (fruit), No (tea)
Rosaceae								
*Thymus linearis* Benth. (127211)	Marveiy	Leaves	June-September	Waste land	Women, men, kids	The leaves are used as herbal tea.	0.09	Yes
Lamiaceae								
*Tragopogon gracilis* D.Don (127167)	Shabey	Aerial parts	July	Fertile valley bottoms	Kids	Directly consumed.	0.12	No
Asteraceae								
*Tulipa lehmanniana* Merckl. (127148)	Sondai	Bulb	April-June	Mountain plateaus	Men, kids	Directly consumed.	0.27	Yes
Liliaceae								
*Viburnum cotinifolium* D. Don (127210)	Thorayi	Fruit	August	Mountain forests	Women, men, kids	Directly consumed.	0.56	Yes
Adoxaceae								
*Viscum cruciatum Sieber* ex Boiss. (127269)	Da shwawna lewanai	Leaves	May	Parasite of olive trees	Women, men	The leaves are used as tea.	0.11	No
Santalaceae								
*Vitis flexuosa* Thunb. (127270)	Malavo	Fruit	June	Hedgerows	Women, men, kids	Directly consumed.	0.02	Yes
Vitaceae								
*Ziziphus jujuba* Mill. (127153)	Bera	Fruit	July	Low land plains	Men, kids	Directly consumed.	0.13	Yes
Rhamnaceae								
*Ziziphus nummularia* (Burm.f.) Wight & Arn. (127271)	Karkanr	Fruit	July	Low land plains	Kids	Directly consumed.	0.01	Yes
Rhamnaceae								
*Ziziphus oxyphylla* Edgew. (127199)	Heilaneiy	Fruit	July	Forests	Women, men, kids	Directly consumed.	0.28	Yes
Rhamnaceae								

### Wild food plant categories

The WFPs were categorized into three groups, i.e. wild fruits (56 %); wild vegetables (33 %) and wild tea (11 %). In the study area, wild fruits were mostly collected by boys below 25 years of age. It has been previously reported that fruits are a highly consumed food category among WFPs [20, 55, 56]. Wild fruits serve as an alternative to cultivated food during dry seasons and times of famine. In most rural communities of developing countries, wild fruits are the only fruits which are consumed by the local people as they cannot afford to buy commercial fruits [57]. In the present study, the number of wild vegetable species was less compared to other ethnobotanical studies [54, 58]. This might be attributed to less annual rainfall [28], insufficient ingredients used in cooking, and excessive use of dairy products among local communities. Similarly, in the study area, the use of wild vegetables was common among communities residing in the foothills, whereas those with large herd sizes migrated to the mountains and preferred dairy products. The lesser importance of wild vegetables in pastoral communities has also been reported by [59]. Although the use of wild tea species was presently uncommon among local inhabitants, their use was documented as this knowledge is vanishing. Inhabitants of the area use 6 species as alternative teas when commercial teas are unavailable or found only at great distances from their settlements.

### Diversity and variation in folk plant knowledge

**Fig. 3:**
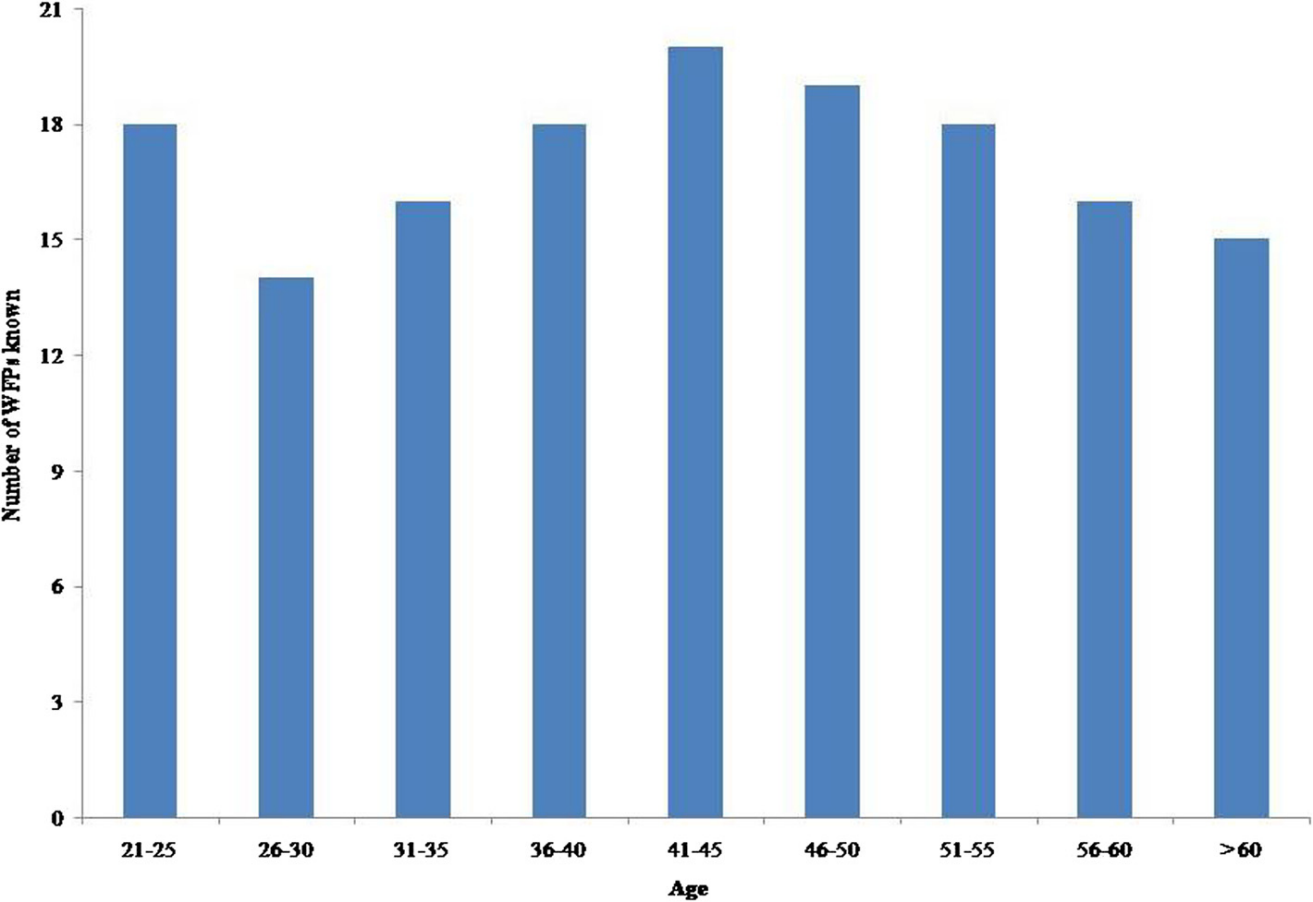
Inter-generational variation of folk wild food plant knowledge recorded in the study area

In the study area, the transfer of WFP knowledge was from parents to children (vertical) as well as within the community (horizontal), but weaker contacts were developed among different villages. In total, 47, 40, 36, 24 and 24 plant species were documented in Landi Kutherzai, Payor Mela, Jatty Ghbaz, Zindawar and Kurachai villages, respectively. Of these, 31 % (16 spp.) were known to all foothill village informants, while 18 % (9 spp.) were only known in a single village. Among the remaining plant species, 24, 12 and 16 % were common to 4, 3 and 2 villages respectively. Village size, exposure to different ecological zones (seasonal migration–see Table 1) and surrounding flora (See Table 2) may contribute to this variation. For example, Landi Kutherzai and Payor Mela inhabitants which migrate between the foothills and the mountains are exposed to both types of edible floras and thus have more knowledge than the other villagers. The inter- and intra-cultural variations of traditional knowledge are quite helpful in understanding its dynamic nature and patterns of development [60]. Each informant mentioned an average of 17.5 species (11 wild fruits, 6 wild vegetables, and 1.5 wild tea species). Figure 3 depicts the disparities in knowledge of different age groups on the basis of 5 years age differences. The age group 41–45 was more knowledgeable as a result of firsthand experience. Age as an influencing factor on WFP knowledge has also been mentioned in the literature [61]. However, the decrease of knowledge exhibited by the +45 age groups was the result of a reduction in their direct involvement in the collection and consumption of WFPs. Interestingly, the 21–25 years age group showed good traditional knowledge which is a sign of traditional knowledge consistency in the area. These individuals are involved in livestock herding during which they not only eat WFPs, but also collect them for later consumption at home. Younger people with considerable knowledge of WFPs (as a result of similar behaviors) have also been reported by Uprety et al. [20] and others [62, 63]. The minor folk knowledge retained by the age group 26–30 can be explained by the fact that these age group members sometimes left their villages and migrated for labor to towns or overseas to support their families and came back to the villages later. Studying individual ethnobotanical knowledge has the potential to add to the systematic understanding of humanity’s most prevalent and earliest form of knowledge [60].

### WFPs storage and their perceived medicinal values

**Fig. 4:**
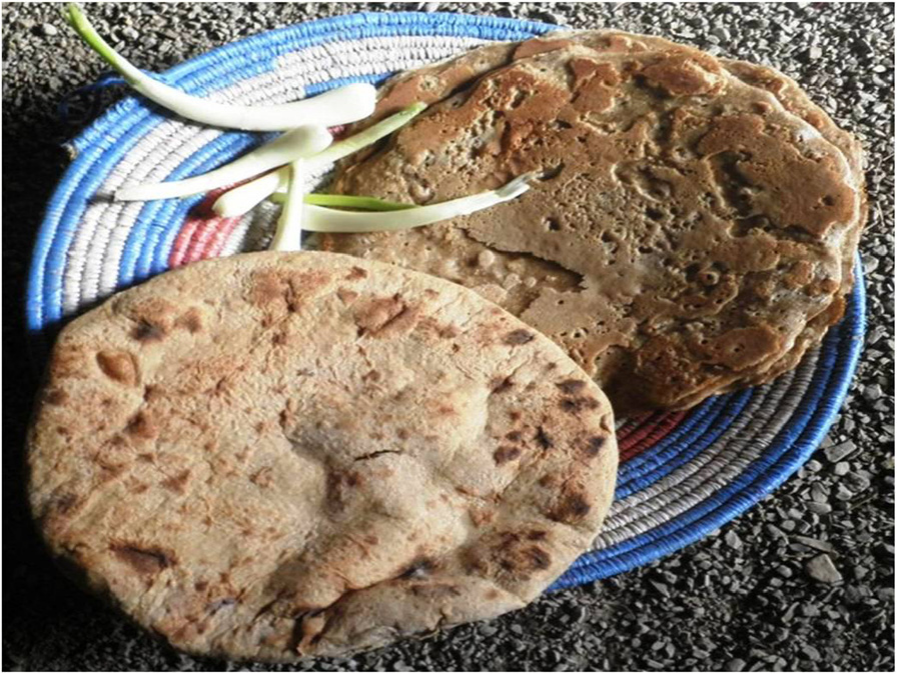
Wild *Allium* sp. eaten with bread in a traditional lunch

The offseason availability of WFPs through drying and storage are valuable practices as they provide food stability and therefore food security throughout the year [64]. Inhabitants of the study area store 16 % (8) of WFP species, including *Allium* sp. (Fig. 4), *Olea ferruginea*, and *Sideroxylon mascatense* for use during the winter season; therefore, these species have significantly higher FC values than non-storable species (Mann–Whitney-*U*-Test for independent samples, *p* < 0.0001). We found that 29 % (14 spp.) were exploited for additional cultural uses, for example, as fodder (*Grewia tenax*, *G. villosa*, *Celtis australis*), fuel and timber (*Pinus wallichiana*, *P. gerardiana*), as well as for multiple purposes (*Olea ferruginea* and *Sideroxylon mascatense*). The additional qualities of WFPs enhance their local preference as significantly higher FC values were recorded for such species (Mann–Whitney-*U*-Test for independent samples, *p* = 0.016). Furthermore, significantly higher FC values were also obtained for species with medicinal uses (Mann–Whitney-*U*-Test for independent samples, *p* = 0.001). The present results follow the general rule that the more versatile a plant, the more widespread its usefulness [65]. The medicinal importance of WFPs (given their role in the prevention of illnesses) has been explained in detail by [3, 15]. The manifold uses of these plants demonstrate their local importance for subsistence and as cultural heritage [66, 67].

### Trade, domestication, and conservation of WFPs

**Fig. 5:**
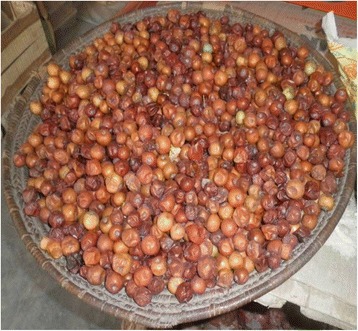
Wild *Ziziphus jujuba* sold by local venders in the study area

In our documentation of WFPs, we observed that 14 % (7species) were sold in markets. The species with marketable potential, such as the species *Pinus gerardiana, Caralluma tuberculate,* and *Ziziphus jujube* (Fig. 5) were more important in the minds of local inhabitants (Mann–Whitney-*U*-Test for independent samples, *p* = 0.002), as they provide opportunities for supplementing family income and such species have attracted worldwide attention [12]. Among all WFPs, 16 % (8 species) were rarely available in the study area. The unavailability of these species was generally due to the unsuitable edaphic and climatic conditions (*Lactuca dissecta*, *Notholirion thomsonianum*, *Pistacia khinjuk*), although over-exploitation for marketing purposes was also responsible (*Caralluma tuberculata*). Sustainable use of such economically important species, however, could make a valuable contribution to the income of indigenous communities [68, 69]. Additionally, it is believed that food botanical studies are vital for the conservation of cultural history, local identity and tradition dishes of a region [27]. The importance of traditional ecological knowledge in the conservation of biodiversity has been recognized by the United Nations Convention on Biological Diversity that calls for the recognition and protection of traditional knowledge [70]. In this study, 14 % (7 species) of WFPs were semi-cultivated (i.e. present in both wild and cultivated forms), including *Ficus palmata* and *Punica granatum*. The domestication of species is the most important cultural development in the past 13,000 years of human history [14]. The wild species of *Allium, Punica granatum, Cicer nuristanicum* and *Ficus palmata* collected from the present study area may be quite useful for genetic cross-over with their domesticated sister species in order to not only improve crop yields, nutrient levels, disease resistance, and the ability to withstand adverse climate change, but also broaden the genetic diversity of present crops to fulfill the requirements of the 21st Century [10, 11, 71].

### Seasonal variations in the ecological availability of WFPs

The seasonality patterns of WFPs demonstrate that May, June, July, and August are regarded as the peak months, during which 33 % (17 spp.), 37 % (19 spp.), 39 % (20 spp.) and 25 % (13 spp.) are obtained, respectively. Likewise, January, February, November, and December are included as off months in which no WFPs are obtained. For fruits and vegetables the peak months are July, October and June, in that order. Similarly, the duration of availability of fruits is generally about only a month whereas for vegetables it lasts 2–5 months (Table 3). Overall, the majority of the species are available during the summer, i.e. hot-dry season which correlates well with shortages of cultivated food resources. The same phenomenon has been reported by Campbe [72], who explained that people need supplementary foods during periods of food shortages. The supply chain in the area was short (except for *Pinus gerardinana*) with collectors/wholesalers selling either to retailers or directly to consumers. The collectors/wholesalers (mostly young boys) were responsible for bringing the WFPs to the market with little or no additional costs, where retailers and consumers can purchase them. The same details are mentioned by Karaan et al. [73] in relation to the marketing and supply chain of indigenous fruit markets in Tanzania, Zimbabwe and Zambia.

### Cultural value of WFPs

Multiple methods of data collection were used (as explained above) for understanding the characteristics of WFPs. Detailed interviews demonstrate that *Pinus gerardiana* is the most important species in the area; however its FC value does not convey its actual importance. The reason for this is that *Pinus gerardiana *is not merely a WFP in the minds of locals; it also makes a tremendous contribution to their livelihood. Similarly, *Punica granatum* was appreciated as a quite important fruit in detailed interviews but its FC value does not represent this. *Punica granatum* is regarded less as a wild plant and more as a cultivated one, which causes it to be less cited. Conversely, *Notholirion thomsonianum* was recorded with a high FC value (0.31) in freelisting but less attention was given during the detailed interviews. On the other hand, the interviews suggested that wild tea species have no importance in the area but its separate freelisting made it a category that highly influenced their FC values. Despite the importance of different quantitative tools, semi-structured, detailed interviews are the best way of exploring the actual importance of ethnobotanical species [65]. Reyes-García et al. [74] stated that the value obtained through a freelisting does not necessarily correspond to its practical value as they found that some species frequently mentioned in freelisting exercises were rarely used. Multiple methods have to be employed for determining the present uses of plants as different indices do not, in all cases, measure the plants’ uses but rather their knowledge [65].

### Collection sites

This study investigated foothill and mountain villages that were separated on the basis of altitude, flora, rainfall, diversity and density of vegetation. This study reports a significantly higher number of species 61 % (31 spp.) in the foothills as compared to the mountains 39 % (20 spp.); however, the FC values of mountain species were significantly higher than for those in the foothills. A large proportion of informants do not migrate to the mountains, and as a result they remain unaware of the edible flora of these areas. Previous studies have mentioned that convenience and experience make the immediate vicinity a favorable collection site [55]. Moreover, the use of wild plants is primarily based on the utilization of species belonging to the closest ecological environments [75, 76]. Two possible factors may account for the high FC values: the selection of the best species among the diverse flora in the mountains and more dependence on natural food entities due to the inaccessibility of markets. Furthermore, there was a prevailing local belief that the mountains have more spiritual potential than the foothills which may influence local inhabitants to give more preference to mountainous species. Weckerle et al. [55], while examining ritual species in the Hengduan Mountains of Southwest China, explained the local perception regarding the availability of the best quality ritual species in high mountains due to a strong association of the mountains with god.

### Comparison with Pakistani ethnobotanical data

To the best of our knowledge, after an extensive literature survey [42, 47–53, 58, 77–131], 73 % (37) species recorded in this study had previously been reported by various investigations in Pakistan, whereas 27 % (14 spp.) are newly reported WFPs and thus represent an addition to the knowledge of the wild edible flora of the country. The average FC values of the newly reported WFP species were significantly lower than the overall FC, which indicates that they are not very important in the area. However, some species which do have high FC values and thus represent important WFPs, such as *Salvia moorcroftiana* (FC: 0.47), *Notholirion thomsonianum* (FC: 0.31) and *Cotoneaster pruinosus* (FC: 0.22), had not yet been reported for the country. Several studies reported the use of these species for different purposes in other areas, including the medicinal use of *Salvia moorcroftiana* [77, 78, 132, 133] and *Notholirion thomsonianum* [125]. This may probably be due to specific cultural knowledge of each community, a phenomena that has been reported in other studies [45, 75]. Some species such as *Viscum cruciatum*, *Cicer nuristanicum* and *Berberis calliobotrys* may not have been reported in other areas due to their restricted occurrence in the country [134]. The newly documented species were collected from diverse habitats ranging from waste land to pine forests, by different collectors (women, men, kids), and during different seasons (Table 3). Moreover, they represented a broad range of WFPs including wild fruits (*Berberis calliobotrys, Cotoneaster pruinosus*), wild culinary vegetables (*Notholirion thomsonianum*, *Cynoglossum lanceolatum*), wild salad greens (*Cirsium arvense*, *Salvia moorcroftiana*), wild tea species (*Limonium cabulicum*, *Spiraea canescens*), and wild relatives of cash crops (*Cicer nuristanicum*, *Allium* sp.). This information may broaden the existing wild food spectra and might provide insight into food diversification. In the context of the recent rapid decline in traditional knowledge across the globe [135–137], exploration of new WFPs is quite encouraging.

## Conclusions

The present study indicates that WFPs still represent an important part of the local food culture in the Thakht-e-Sulaiman Hills of NW Pakistan. The knowledge of such species, although persistent, is declining. Exposure to wider ecological zones, the surrounding vegetation and age of the inhabitant were all factors influencing the extent of traditional knowledge of WFPs. In addition to food value, the supplementary qualities of WFPs such as medicinal potential, cultural uses, marketing and storage make them more important in the local culture but also predispose them to extensive exploitation. There is a large potential for the harvesting, domestication and marketing of WFPs in the area, and if done properly, they could be a source of cash income for locals. The wild relatives of the domesticated food species could help increase genetic diversity for crop improvement and yield, thus addressing the present demand of human food security. The ongoing process of domestication of wild species in the area is of the utmost importance not only for the interests of local communities but also for global food diversification.
